# Leucine Supplementation Improves Effort Tolerance of Rats With Hyperthyroidism

**DOI:** 10.3389/fphys.2018.01632

**Published:** 2018-11-20

**Authors:** Thiago Montes Fidale, Hanna Karen Moreira Antunes, Leonardo Roever, Alexandre Gonçalves, Guilherme Morais Puga, Romeu Paulo Martins Silva, Fernando Nazário de Resende, Fernanda Rodrigues de Souza, Beatriz Montes Fidale, Frederico Balbino Lizardo, Elmiro Santos Resende

**Affiliations:** ^1^Laboratory of Experimental Medicine, Federal University of Uberlândia, Uberlândia, Brazil; ^2^Special Academic Unit of Biotechnology, Federal University of Goiás, Catalão, Brazil; ^3^Post-graduate Science in Health, Federal University of Uberlândia, Uberlândia, Brazil; ^4^Department of Biosciences, Federal University of São Paulo, São Paulo, Brazil; ^5^Master Institute of Education, IMEPAC, Araguari, Brazil; ^6^Faculty of Physical Education, Federal University of Uberlândia, Uberlândia, Brazil; ^7^College of Physical Education, Federal University of Acre, Acre, Brazil; ^8^Institute of Biomedical Sciences, Federal University of Uberlândia, Uberlândia, Brazil

**Keywords:** creatine kinase, thyrotoxicosis, BCAA, Wistar, exercise

## Abstract

Leucine is a regulator of protein metabolism *in vivo* and information on its action on effort tolerance of both animals and humans with hyperthyroidism is scarce. The objective of the present study was to verify the influence of leucine supplementation on the effort tolerance of Wistar rats with experimental hyperthyroidism. 40 animals were divided into four groups of ten: control (C), hormone (H), leucine (L), and hormone + leucine (HL). Hyperthyroidism was induced by daily administration of 20 μ⋅g100 g^-1^ of levothyroxine sodium in aqueous suspension by gavage. Leucine was supplemented by adding 5% of the amino acid to the conventional feed. The animals’ blood was collected by cardiac puncture to analyze TSH, T4, and T3 levels. The effort tolerance was determined by the swimming test with a 7% load attached to animals’ tails. Statistical analysis was performed using the Shapiro-Wilk normality test, followed by the analysis of variance (ANOVA) of repeated measures of two factors (treatment × time) and Tukey *post hoc*, with a significance level of *p* < 0.05. Administering thyroid hormone increased the swimming performance of rats after 14 and 21 days, but with a drop in performance at 28 days. The HL group, on the other hand, had a significantly higher swimming performance compared to the other groups after 28 days of treatment. Leucine supplementation associated with the experimental model of hyperthyroidism improved the performance of rats in a swimming test after 28 days of treatment.

## Introduction

The physiological effects of thyroid hormones ultimately result from the nuclear transcription of large numbers of genes, causing a generalized increase in functional activity throughout the body, metabolic rate of all body tissues, the basal intensity of O_2_ consumption, and the production of heat. Thyroid hormones can raise metabolism up to 100% above normal and the effects include increased heart rate, cardiac output, and decreased systemic vascular resistance, among others (Klein and Ojamaa, 1998). Progressive muscular weakness associated with a generalized muscular atrophy occurs in patients with hyperthyroidism, thus compromising the quality of life and the ability to accomplish daily tasks (Dillmann, 2010).

In this context, leucine is an amino acid of the branched chain amino acid (BCAA) group that regulates muscle protein metabolism *in vivo* (Kobayashi et al., 2006). Leucine supplementation has been used as a nutritional strategy to treat muscular disorders induced by several clinical disorders (Eley et al., 2007; Han et al., 2007; Fidale et al., 2018).

Pioneering studies by Shah et al. (2000) demonstrated that leucine supplementation in rats is able to markedly stimulate protein synthesis in skeletal muscle, activating the mTOR intracellular signaling pathway. Martin et al. (2017) observed in a cell culture model of skeletal muscle tissue that leucine improves mTOR signaling, associated with microtubule hypertrophy and increased maximal contractile force by electrical stimulation, providing evidence for the efficacy of leucine as an anabolic nutritional agent which may influence the functional capacity of muscle.

Leucine modulates protein synthesis by increasing post-transcription efficiency, enhancing the translation rate of mRNAs (Kimball and Jefferson, 2006; Wilkinson et al., 2017). The mechanism of synthesis is due to phosphorylation of the protein kinase p70S6k, which induces the phosphorylation of the ribosomal protein (S6), the eukaryotic initiation factor (eIF4B) and a protein involved in stretching the translation process, Eukaryotic elongation factor kinase 2 (eEF2k), which affects the initiation and elongation of other mRNA classes (Ananieva et al., 2016; Lane et al., 2017).

The hyperthyroidism chronically erating intolerance to the physiological physics in patients, however, uncertainties about the action of leucine on effort tolerance in hyperthyroidism. Therefore, the hypothesis of the present study was to test an experimental model for a period of 28 days from the time we performed a study on the performance of rats, as well as maintaining that performance in leucine supplementation. The present study aimed to verify the efficacy of leucine supplementation in an experimental model of hyperthyroidism in the effort tolerance of Wistar rats.

## Materials and Methods

The present study was approved by the Ethics Committee on the Use of Animals of the Federal University of Uberlândia (CEUA-UFU), under opinion number 193/11. For the study, 40 male Wistar rats at 10 weeks old and 370 ± 12 g mean weight were used. The experiment was carried out in the UFU experimental medicine laboratory and had a total duration of 35 days: 7 days of adaptation to the laboratory and 28 days of the experiment. During the experimental period, the ambient conditions of the laboratory were constant with respect to temperature, noise level, and brightness, with a 12-h light and dark cycle.

The animals were randomly divided into four groups of 10 animals each, control group (C), hormone group (H), hormone + leucine group (HL), and leucine group (L). The rats were kept separate in collective boxes with five animals per box.

All animals had free access to water and feed, animal body weight, feed intake, water, as well as fecal and urinary volume were observed during 2 weekly times stipulated according to laboratory standards. All experimental procedures were performed in strict accordance with the international regulation on animal welfare.

### Leucine Supplementation

The standard diet presented a minimum concentration of 1.54 g⋅100 g (1.5%) of leucine, according to the American Institute of Nutrition (AIN-93G) which was provided to both control and hormone groups. For the leucine and leucine + hormone groups treated with a leucine-supplemented diet, a standard diet, plus 5.0 g⋅100 g^-1^ (6.5% total) leucine of the total dietary nutrients, which was previously used by Witham et al. (2013).

### Experimental Hyperthyroidism

The animals of group H and HL received a daily dose of 20 μg⋅100 g^-1^ in aqueous suspension, at2 mL⋅kg^-1^ of 0.01% T4 during the 28-day experimental period (Engelman et al., 2001; Fernandes et al., 2007). Animals ingroups C and L received a similar dose of saline solution in the same regimen used for hormone-treated animals.

### Effort Test

**FIGURE 1:**
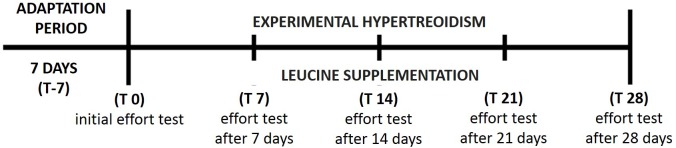
Schematic drawing of the experimental protocol, with T-7, 7 days of adaptation to laboratory environment; HE, experimental hyperthyroidism; SL, supplementation with leucine; T0, initial effort test at last day of the adaptation week; T7, effort test after 7 days of experimental protocol; T14, effort test on the 14th day of the experimental protocol; T21, effort test on the 21st day of the experimental protocol; T28, effort test on the 28th day of the experimental protocol, weighing, blood collection, and euthanasia of the animals.

All animals were subjected to swimming tests and the intensity of the load was 7.0% of rat body weight, according to Prada et al. (2004). This load was attached to the tail of the animal, and it was placed in an individual tank with water in which it swam to exhaustion. The tests were performed on the last day of the adaptation week and repeated every 7 days until the end of the experiment according to Figure 1.

The temperature of the water was maintained between 30 and 32°C because it was considered thermally neutral in relation to the body temperature of the rat (Harri and Kuusela, 1986).

### Euthanasia of Animals and Blood Collection

After the last effort test (day 28), all the animals were kept at CEBEA-UFU for 24 h, with free access to feed and water. According to Longley (2008), fasting before euthanasia is unnecessary for rodents, since they do not vomit, and present high metabolic rates in addition to storing little glycogen in the liver, which could lead to hypoglycemia associated with experimental hyperthyroidism. This practice complies with the guidelines of the National Animal Experimentation Control Council (Ministry of Science, Technology and Innovation and National Council for Control of Animal Experimentation (CONCEA), 2018). Later, the animals were anesthetized for blood collection through cardiac puncture for the determination of triiodothyronine (T3), tetraiodothyronine (T4), and thyroid stimulating hormone (TSH) levels using INTERKIT^®^ and for the quantification of Creatine Kinase Myocardial isoenzyme (CK-MB) was used the Liquiform 60 ml KIT of LABTEST^®^. The analysis was done using the enzyme-linked immunosorbent assay (ELISA) method, using the Biochemical Analyzer (LABMAX PLENNO^®^) Serial No. 1308.18 of the Laboratory of Veterinary Medicine of the Federal University of Uberlândia.

### Statistical Analysis

Statistical analysis was performed using the Shapiro-Wilk normality test, followed by the analysis of variance (ANOVA) of repeated measures of two factors (treatment × time) and Tukey *post hoc*. Statistical analysis was done with a GraphPad statistical package Prism (5.0 version). Statistical significance was established when the value of *p* < 0.05.

## Results

### General Observations

No deaths occurred in any of the groups during the experiment.

### Effects of High Dietary Leucine on the Analyzed Blood Variables of Wistar Rats in Experimental Hyperthyroidism

Statistically significant differences in TSH and T4 were observed in groups H and HL compared to groups C and L, as shown in Table 1 demonstrating that the experimental model effectively induced hyperthyroidism. Reduction in CKMB concentrations in the L and HL groups was observed, possibly due to reduction of autophagy and anticatabolic action.

**Table 1:** Analyzed variables: Experimental model of hyperthyroidism promoted a significant increase in T4 values and a decrease inTSH values in the H and HL E groups and leucine supplementation reduced CK-MB concentrations in the L and HL groups.

Analyzed Variables		Control	Hormone	Hormone + Leucine	Leucine
Anthropometric Variables	Initial BW (g)	326,2 ± 15,89	305,4 ± 23,61	301,4 ± 9,55	312,0 ± 9,89
	Final BW (g)	348,2 ± 7,32	328,8 ± 22,86	334,8 ± 12,66	328,2 ± 10,44
Feed consumption	FC (g)	32 ± 3	36 ± 2	37 ± 4	32 ± 6
Blood variables analyzed	T3 (ng⋅mL^-1^)	1.98 ± 0.5	2.29 ± 0.9	2.31 ± 0.6	2.06 ± 0.5
	T4 (μg⋅dL^-1^)	4.76 ± 0.8	12.56 ± 3.4^∗†^	12.46 ± 2.3^∗†^	5.09 ± 1.2
	TSH (ng⋅mL^-1^)	1.45 ± 0.35	0.39 ± 0.07^∗†^	0.42 ± 0.08^∗†^	1.36 ± 0.29
	CK-MB (U/L)	1005,75 ± 314	934,31 ± 363	538,75 ± 112^∗^	533,99 ± 43^∗^


*Values expressed in average ± standard deviation for: BW, body weight; FC, feed consumption; T3, triiodothyronine; T4, tetraiodothyronine; TSH, thyroid stimulating hormone; CK-MB, Creatine Kinase Myocardial isoenzyme. ^∗^p < 0.05 compared to group C (ANOVA); ^†^p < 0.05 compared to the L group (ANOVA).*

### Effects of High Dietary Leucine on Effort Test of Wistar Rats in Experimental Hyperthyroidism

Significant results in the effort tolerance of the rats were observed in the third test, performed on the 14th day of the experiment (T14); there were statistically significant differences between group H and groups C, HL, and L, with the highest swimming times presented by the group H in the three analyzed situations. In the fourth effort test (T21), performed at day 21 of the experiment, we also observed significant differences between group H and group C, with the largest swimming time presented by group H.

**FIGURE 2:**
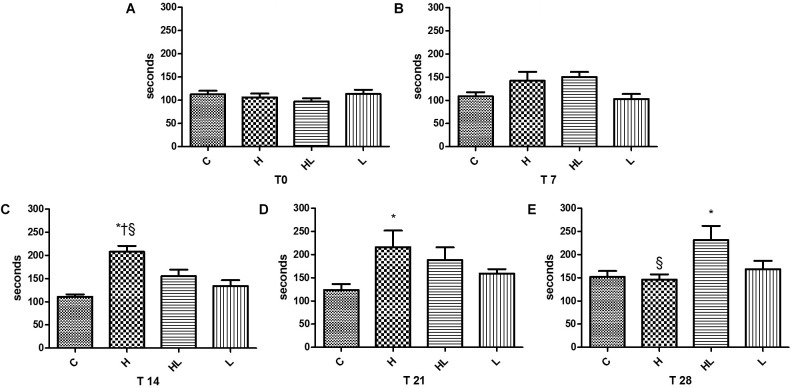
Effort test T0, presented in Graph **A**, performed after the adaptation week; T7 effort test, presented in Graph **B**, performed after 7 days of experiment; T-14 exercise test, presented in Graph **C**, performed after 14 days of experiment, indicating an increase in swimming performance in group H; T-21 exercise test, presented in Graph **D**, performed after 21 days of experiment, evidencing the maintenance of swimming performance gains by group H; Effort test T-28, presented in Graph **E**, performed after 28 days of experiment, showing a decrease in swimming performance by group H and increased performance by the HL group. C, control group; H, hormone group; HL, hormone + leucine group; L, leucine group. ^∗^*p* < 0.05 compared to Group C (ANOVA); ^†^*p* < 0.05 compared to Group L (ANOVA); ^§^ *p* < 0.05 in relation to the HL Group (ANOVA).

In the last test (T28) performed on day 28 of the experiment, upon comparing the HL group to the C group, were observed greater swimming times in the HL group, as shown in Figure 2.

## Discussion

In the present study, after 14 days of experiment, the animals of group H, in experimental hyperthyroidism, presented a longer swimming time when compared to the animals of the other groups, but with a drop in performance in 28 days, being equal to the control group C, according to Figure 2. The addition of leucine to the hormone, suggests a later performance increase. These results suggest a time dependent influence of the hormone on the performance of animals and an influence of leucine at that time.

Thyroid hormones act in virtually all organic systems, with a proven role in metabolism, increased contraction force, myocardial rhythm, and oxidative activity of skeletal muscle (Sun et al., 2000; Sjögren et al., 2007; Simonides and van Hardeveld, 2008). However, prolonged exposure to high doses of thyroid hormone leads to a hypermetabolic state which results in marked loss of body weight, cardiac arrhythmia, degradation of contractile proteins with increased collagen deposition, and a consequent decrease in cardiac function, as well as a marked degradation of energy substrates and skeletal muscle mass.

It was observed in the present study that experimental thyrotoxicosis in rats caused an increase in fecal and urinary volume, requiring a higher frequency in the exchange and hygiene of the animals’ housings, possibly characterizing the hypermetabolic state common to hyperthyroidism. However, there was no significant difference in feed intake and total body weight at the end of the experiment in either group.

All these changes are related to the low tolerance to effort, which has been demonstrated both in animals and in humans exposed to high doses of thyroid hormone (Kahaly et al., 2002; Gonçalves et al., 2006). Martin et al. (1991) investigated the mechanism of reduced effort tolerance in an experimental model of hyperthyroidism, analyzing cardiovascular function and skeletal muscle metabolism in 18 healthy subjects. They observed that experimental hyperthyroidism, induced by daily intake of 100 μg triiodothyronine (T3) for 2 weeks, impaired short-term effort tolerance due to a decrease in skeletal muscle mass and oxidative capacity related to accelerated protein catabolism, without impairing cardiac function.

In the present study, were observed decreased effort tolerance of group H in the swimming test performed on the 28th day (T28) of treatment with thyroid hormone. However, this effect was not as pronounced when the animals’ diets were supplemented with leucine (HL group). Kato et al. (2017) reported that supplementation with essential amino acids enriched with Leucine for 7 days positively modulated glycogen recovery in rat muscle tissue after exercise-induced damage through electrostimulation.

An important finding of the present study was the increased swimming time that the H group presented during the effort tests, swimming 87% more in T14, and 74% more in T21, when compared to group C, but with decreased performance at T28. The results suggest an acute increase in the tolerance to the effort, possibly by positive oxidative adaptations like increased density and mitochondrial activity previously reported by Gustafsson et al. (1965), but the performance decrease at 28 days occurring through damage (Martin et al., 1991; Fidale et al., 2013), and possible reduction of glycogen (Dos Santos et al., 2016).

Kaminsky et al. (1991) correlated thyroid hormone with the action of skeletal muscle and found that it regulates enzymatic activity in aerobic and anaerobic glucose metabolism, which directly influences mitochondrial activity and ATP supply. Argov et al. (1988) observed that both patients and rats with hyperthyroidism did not differ from normal controls during rest and exercise, but they had an unusually rapid recovery after exercise when compared to controls, showing the positive relationship between the hormone and muscular functional activity in this model.

Silva et al. (2015) studied rats treated with thyroxine and observed values of cross-sectional area and creatine kinase (CK) were significantly increased, the authors suggest that thyroid hormones can be used to simulate the adaptations and muscle damage that result from physical exercise.

Blood concentrations of CK are used to help diagnose progressive muscular dystrophy (Ebashi et al., 1959) and variations in CK concentration are an important clinical marker for muscle injury, which is often present during thyroid dysfunction (Rosalki, 1970; Meltezer, 1971).

In the present study were observed that in an experimental model of hyperthyroidism, serum CK-MB concentrations were significantly higher in the hormone group than in the control group, suggesting heart tissue damage possibly due to cardiac stress imposed by the excess of the hormone and myocardial susceptibility to hypoxia. This effect was ameliorated by leucine supplementation, which decreased CK-MB concentration and suggested a protective role of leucine. This protection may be linked to anabolic or anti-catabolic effects on protein metabolism, as reported in previous studies (Canedo et al., 2010; Murphy et al., 2016; Reule et al., 2017).

Despite not finding differences in CK concentrations, Reule et al. (2017) observed greater effort tolerance and strength maintenance in a group of aged, untrained, leucine-supplemented elderly subjects subjected to a fatigue protocol through exercise when compared to the placebo group. These results agree with the findings of the present study, since the group of rats supplemented with leucine did not show decreased performance even in experimental hyperthyroidism, which according to Silva (Argov et al., 1988), can simulate the muscular damage caused by physical exercise.

Intake of leucine-rich protein coupled with a physical exercise program seemed to restore the balance of skeletal muscle protein metabolism by salvaging protein synthesis (Xia et al., 2017).

## Study Limitations

This study presents only preliminary data. Future research is needed to confirm these findings in humans and to elucidate the cellular mechanisms that link leucine to effort tolerance in hyperthyroidism.

## Conclusion

The experimental model of hyperthyroidism increased the swimming time of rats at 14 and 21 days of treatment, but decreased performance at 28 days. Leucine supplementation in an experimental model of hyperthyroidism improved effort tolerance after 28 days of treatment.

## Author Contributions

All authors listed have made a substantial, direct, and intellectual contribution to the work and approved it for publication.

## Conflict of Interest Statement

The authors declare that the research was conducted in the absence of any commercial or financial relationships that could be construed as a potential conflict of interest.
